# A fully haplotype-resolved and nearly gap-free genome assembly of wheat stripe rust fungus

**DOI:** 10.1038/s41597-024-03361-6

**Published:** 2024-05-16

**Authors:** Jierong Wang, Yiwen Xu, Yuxi Peng, Yiping Wang, Zhensheng Kang, Jing Zhao

**Affiliations:** 1 College of Plant Protection, Northwest A&F University, Yangling, Shaanxi 712100 China; 2 State Key Laboratory of Crop Stress Biology for Arid Areas, Northwest A&F University, Yangling, Shaanxi 712100 China; 3 College of Life Science, Northwest A&F University, Yangling, Shaanxi 712100 China

**Keywords:** Structural variation, Agricultural genetics

## Abstract

Stripe rust fungus *Puccinia striiformis* f. sp. *tritici* (*Pst*) is a destructive pathogen of wheat worldwide. *Pst* has a macrocyclic-heteroecious lifecycle, in which one-celled urediniospores are dikaryotic, each nucleus containing one haploid genome. We successfully generated the first fully haplotype-resolved and nearly gap-free chromosome-scale genome assembly of *Pst* by combining PacBio HiFi sequencing and trio-binning strategy. The genome size of the two haploid assemblies was 75.59 Mb and 75.91 Mb with contig N50 of 4.17 Mb and 4.60 Mb, and both had 18 pseudochromosomes. The high consensus quality values of 55.57 and 59.02 for both haplotypes confirmed the correctness of the assembly. Of the total 18 chromosomes, 15 and 16 were gapless while there were only five and two gaps for the remaining chromosomes of the two haplotypes, respectively. In total, 15,046 and 15,050 protein-coding genes were predicted for the two haplotypes, and the complete BUSCO scores achieved 97.7% and 97.9%, respectively. The genome will lay the foundation for further research on genetic variations and the evolution of rust fungi.

## Background & Summary

The basidiomycete fungus *Puccinia striiformis* f. sp. *tritici* (*Pst*) is an obligate biotrophic pathogen that causes stripe (yellow) rust disease in wheat. Stripe rust has been reported in more than 60 countries, threatening 88% of wheat production worldwide and seriously affecting the global food supply^1–3^. The damage of this pathogen to agriculture is attributed to its massive genetic diversity because of sexual recombination mainly occurring in the Himalayan and neighboring regions (Nepal, Pakistan, and China), its long-distance dispersal across continents by means of nature and human transport, and its fast local adaptation through stepwise mutation and somatic hybridization, surmount the resistance of wheat cultivars and result in subsequent epidemics^4–8^. As a macrocyclic and heteroecious rust fungus, *Pst* has an extremely complex lifecycle, comprising five different types of spores (urediniospores, teliospores, basidiospores, pycniospores, and aeciospores) on two phylogenetically unrelated plant hosts: wheat is the primary host and barberry (*Berberis* spp.) is the alternate host^9^. The threat to wheat arises from urediniospores re-infecting and exponentially multiplying through the asexual cycle during the wheat growing season. The one-celled urediniospore is dikaryotic (*N + N’*), with a full set of haploid chromosomes in each separate nucleus (karyon), and is highly heterozygous^10–12^. Therefore, a high-quality haplotype-resolved genome assembly in nonhaploid rust fungi is important for in-depth research on genetic variation within and across species.

Although a haplotype-phased chromosome-scale genome of *Pst* has been reported, it has not been completely resolved and hundreds of gaps remain^13^. With the advancement of sequencing technologies and bioinformatics software, more and more complex genomes of animals and plants have achieved haplotype-resolved and telomere-to-telomere (T2T) construction^14–17^. Currently, PacBio High-Fidelity (HiFi) sequencing technology yields long reads averaging 10–25 kb and extremely low error rates (<0.5%), which are the main data types for high-quality genome assembly^18,19^. Furthermore, the trio-binning assembly strategy using short reads from two parental genomes provides a perfect approach for producing a completely haplotype-resolved diploid genome^18,20^. In this study, we combined PacBio HiFi sequencing technology and a trio-binning approach to obtain two primary haploid assemblies of the *Pst* isolate AZ2, which was derived from the *Pst* isolate A153 crossing with isolate XZ-2. Next, high-throughput chromosome conformation capture (Hi-C) sequencing technology was applied to scaffold the assembled data at the chromosome level. To reduce the influence of heterozygous genomic regions of the parents on haploid phasing, DNA data from haploid pycniospores from parental isolates A153 and XZ-2 were sequenced with single-cell genomic sequencing technology and used to partition HiFi reads into haplotypes.

Here, we successfully generated the first fully haplotype-resolved and nearly gap-free chromosome-scale genome for the dikaryotic wheat stripe rust fungus. The genome size of the two haploid assemblies was 75.59 Mb and 75.91 Mb, with both anchored onto 18 pseudochromosomes. In total, 15 and 16 gapless chromosomes were separately assembled for the two haplotypes, and the other chromosomes each contained only 1–2 gaps. A total of 15,046 and 15,050 protein-coding genes were predicted for the two haplotypes, and the complete BUSCO scores reached 97.7% and 97.9%, respectively. Meanwhile, a complete and circular mitochondrial genome (mitogenome) of *Pst* was also assembled, with a total size of 101,852 bp. Multiple assessment methods have confirmed the high continuity, correctness, and completeness of the haplotype-resolved assembly. This study will be a useful resource for community research on the pathogenicity, genetic variation, and evolution of the *Pst* genome.

## Methods

### Isolate selection and sexual hybridization

Sexual hybridization between *Pst* isolates A153 and XZ-2 was performed based on previously reported procedures^21–23^. When obvious nectars (or honeydews) formed, a partial nectar from one pycnium of A153 or XZ-2 was separately aspirated with a pipette gun for DNA extraction, and the remaining nectar from the same pycnium of A153 was transferred to the same pycnium of XZ-2 for mating and sexual hybridization. The aeciospores generated on the barberry host were collected to inoculate the susceptible wheat cultivar Mingxian 169 seedlings for the production of uredinium. Only a single urediniospore produced on Mingxian 169 was selected to inoculate the seedlings of Mingxian 169 and multiplied, forming the progeny isolate AZ2.

### Genome and transcriptome sequencing

Genomic DNA of AZ2 was extracted from freshly harvested urediniospores using the previously described method^24^. For PacBio HiFi sequencing, an SMRT bell library was constructed and sequenced on the PacBio Sequel II system, and ~9.44 Gb consensus HiFi reads were generated using CCS software with default parameters, to achieve approximately 124 × coverage of the size of the haploid genome. Meanwhile, a DNA library with 350-bp fragment sizes was constructed and sequenced using the Illumina Novaseq PE150 platform, with ~77 × coverage of the haploid genome size. The Hi-C library was constructed using a 4-cutter restriction enzyme *DpnII* with fresh ungerminated AZ2 uredinospores, and ~18.22 Gb reads were generated on the Illumina Novaseq PE150 platform, with ~240 × coverage of the haploid genome size (Table 1).

**Table 1:** Summary of sequencing data of *Puccinia striiformis* f. sp. *tritici* for haplotype-resolved assembly and genome annotation.

Sequencing	Clean base (Gb)	Clean reads	N50 length (bp)	Depth (×)	Sample	Application
HiFi	9.44	705,557	13,655	124.18	urediniospores of AZ2	Genome assembly
Hi-C	18.22	121,470,554	2 × 150	239.74	urediniospores of AZ2	Chromosome construction
Illumina	5.85	39,008,242	2 × 150	76.99	urediniospores of AZ2	Genome evaluation
RNA-seq	8.85	58,986,308	2 × 150	—	ungerminated urediniospores and infected wheat leaves (7 and 9 days post infection) of AZ2	Genome annotation
Single-cell genome	10.06	67,066,358	2 × 150	132.37	pycniospores of A153	Genome assembly
Single-cell genome	10.01	66,740,788	2 × 150	131.73	pycniospores of XZ-2	Genome assembly

AZ2 RNA was extracted separately from fresh urediniospores, 7 days and 9 days after inoculation on the susceptible wheat cultivar Mingxian169 using the Qiagen (Doncaster, Australia) Plant RNeasy kit as previously described^25^. Equal amounts of the three RNA samples were mixed for mRNA sequencing using Illumina Novaseq sequencing, and ~8.85 Gb reads were generated (Table 1). All sequencing studies were carried out at Novogene Corporation (Beijing, China).

### Single-cell genomic sequencing of the pycniospore

The genomic DNA of A153 and XZ-2 from freshly harvested pycniospores was separately prepared and sequenced using single-cell genomic sequencing with multiple displacement amplification, both generating ~10 Gb reads on the Illumina Novaseq platform and achieving ~132 × coverage of the haploid genome size (Table 1). Sequencing was performed at Annoroad Gene Technology Corporation (Beijing, China).

### Genome size and heterozygosity estimation

**Fig. 1:**
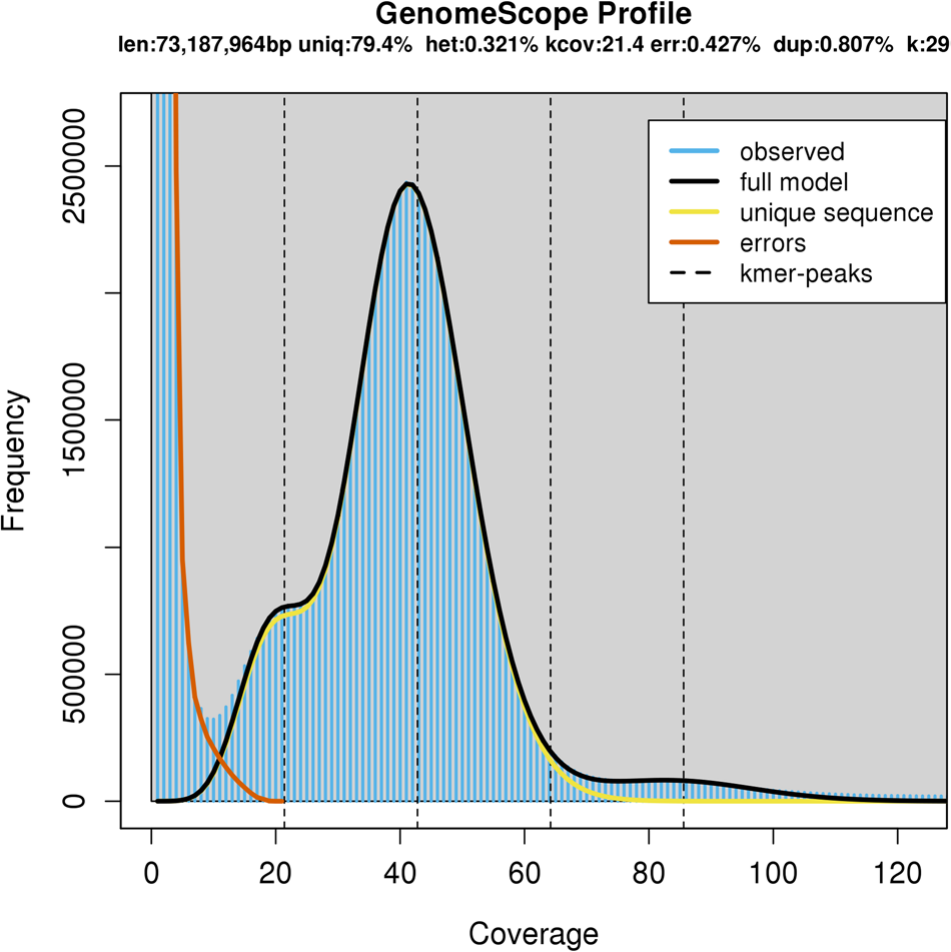
The GenomeScope profle of *Puccinia striiformis* f. sp. *tritici* isolate AZ2 based on 29-mer.

Before assembly, genome size and heterozygosity were estimated with Illumina short DNA reads. Jellyfish v2.3.0^26^ was used to calculate the frequency distribution of the depth of clean data with 29-mer. The results were then imported to GenomeScope v1.0^27^ to estimate the basic features of the genome with 29-mer. The haploid genome size of AZ2 was estimated to be 73.19 Mb, with a heterozygosity rate of 0.32% (Fig. 1).

### Haplotype-resolved genome assembly

PacBio HiFi sequencing technology and a trio-binning strategy^20^ were combined using Hifiasm v0.16.1^28^ with default parameters to generate a haplotype-resolved *Pst* assembly. In the first step, yak v0.1-r56 (https://github.com/lh3/yak) was used to count 19-mer with the Illumina short reads from pycniospores of the paternal isolate A153 and maternal isolate XZ-2. Next, HiFi reads from AZ2 were partitioned into haplotype-specific sets using parental sequencing data and subsequently assembled, respectively. Clean Hi-C paired-end reads were aligned with the assembly using Juicer v1.6.2^29^ with the BWA algorithm to obtain the interaction matrix. The 3d-DNA v180922 pipeline^30^ was applied to reorder and scaffold the contigs. The position of the contigs was also manually adjusted based on the Hi-C heatmaps visualized using JuicerBox v1.9.8^31^. Blastn searches against the NCBI nr/nt database were used to check potential contamination and none of the contigs had significant hits to noneukaryotic sequences, chloroplast sequences, mitochondrial sequences, or plant rRNA with E-value set as 1e-10. The obtained contigs were parsed by Purge Haplotigs v1.1.1^32^ and Redundans^33^ to eliminate the redundancies.

**Fig. 2:**
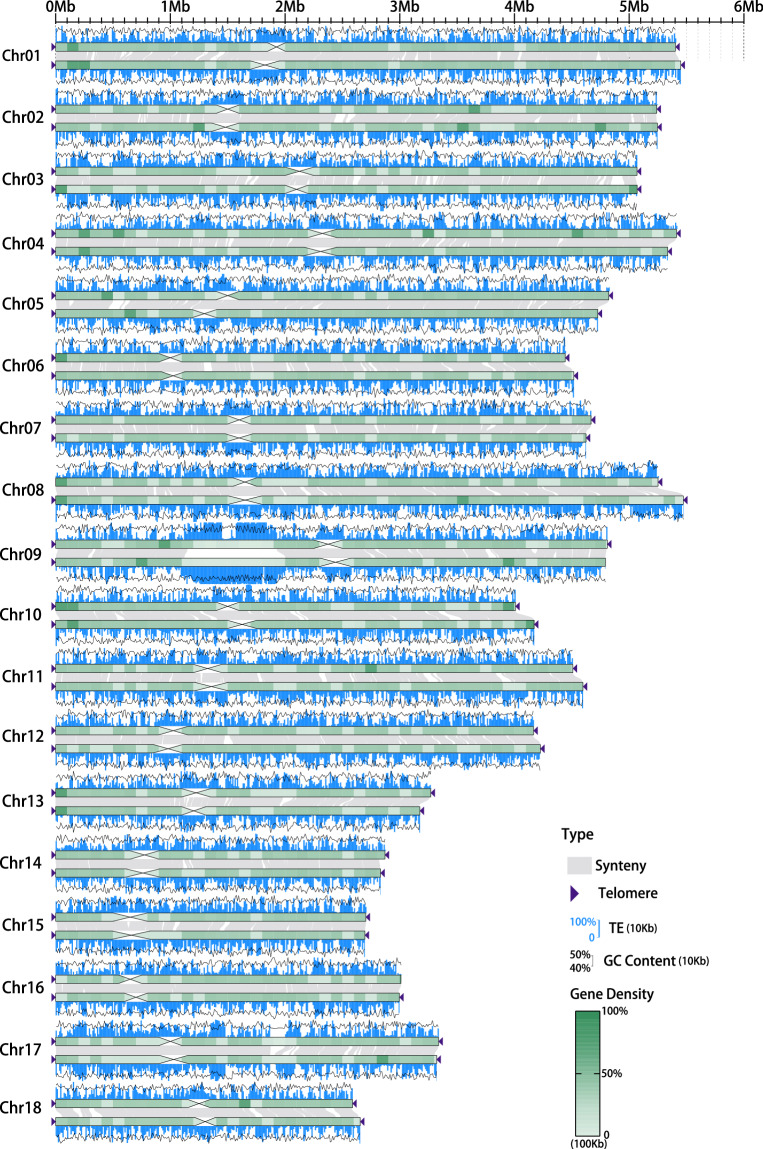
Overview of the haplotype-resolved genome assembly of *Puccinia striiformis* f. sp. *tritici* isolate AZ2. All 18 chromosomes of the AZ2 are drawn to scale and the ruler indicates chromosome length. Collinear regions between the two haplotypes are shown by gray lines. The cross-like shapes indicate the positions of the centromeres. The deep purple triangles indicate the presence of telomere sequence repeats.

The final assembled genome contained two fully separated haplotypes, named AZ2A (75.59 Mb) and AZ2B (75.91 Mb), both with 18 pseudochromosomes (Table 2, Fig. 2). The genome size previously estimated using the k-mer frequency was similar to that of these assemblies. The contig N50 length of the two haplotypes was 4.17 Mb and 4.60 Mb, respectively. Remarkably, of the total 18 chromosomes, 15 and 16 were gapless while there were only five and two gaps for the remaining chromosomes of the two assembled haplotypes, respectively (Supplementary Table 1), suggesting good continuity of the genome assembly.

**Table 2:** Summary of *Puccinia striiformis* f. sp. *tritici* isolate AZ2 genome assembly data.

Statistic	AZ2A		AZ2B	
Total sequence length (bp)	75,586,642		75,908,592	
Number of gaps	5		2	
Total assembly gap length (bp)	500		200	
GC content (%)	44.43		44.44	
Number of telomeres	34		35	
**Characteristic**	**Contig**	**Scaffold**	**Contig**	**Scaffold**
Number	23	18	20	18
Max. (bp)	5,404,001	5,412,896	5,471,935	5,471,935
Min. (bp)	770,784	2,587,901	1,438,225	2,655,380
Mean. (bp)	3,286,354	4,199,258	3,795,420	4,217,144
N50 (bp)	4,166,297	4,668,497	4,597,639	4,623,987

### Repeat and gene annotation

RepeatModeler v1.0.8 (https://www.repeatmasker.org/RepeatModeler/) constructed a *de novo* repeat library, which was then merged with Repbase library v23.09 (https://www.girinst.org/repbase/) and imported it into RepeatMasker v4.1.2-p1^34^ for repeat prediction. A total of 27.88 and 28.38 Mb of repetitive sequences were identified, accounting for 36.89% of AZ2A and 37.39% of AZ2B, of which long terminal repeats (LTR) and DNA elements were the abundant repetitive elements despite unclassified repeats (Fig. 2, Supplementary Table 2).

The genome of repeats soft-masked was used for gene annotation using the funannotate pipeline (https://github.com/nextgenusfs/funannotate). Clean RNA-seq reads from AZ2 were aligned to the genome using Hisat2 v2.2.1^35^ with ‘–max-intronlen 10000’, ‘–min-intronlen 20’ and default parameters for training gene models. The EST clusters of *Pucciniamycotina* were downloaded from the JGI MycoCosm website (http://genome.jgi.doe.gov/pucciniomycotina/pucciniomycotina.info.html) and used as transcript evidence. Proteins from previous *Pst* studies including Pst-104E^36^, Pst-DK0911^37^, Pst93-210^38^, CYR34^38^ and Pst-134E^13^ were combined with the default UniProtKb/SwissProt curated protein database of funannotate as protein evidence. Genes were predicted using a suite of funannotate pipeline tools, including Augustus v3.3.3^39^, GeneMark-ES v4.32^40^, CodingQuarry v2.0^41^, SNAP v2006-07-28^42^ and GlimmerHMM v3.0.4^43^. All the above gene models were combined using EvidenceModeler v.1.1.1^44^ with default weight settings. A total of 15,046 and 15,050 protein-coding genes were predicted for AZ2A and AZ2B, respectively. The total lengths of the protein-coding genes were 23.93 Mb and 24.05 Mb, respectively (Table 3, Fig. 2). The mean lengths of the genes were 1.59 kb and 1.60 kb. There were 12,872 and 12,883 genes with an additional exon.

**Table 3:** Statistics of protein-coding genes in AZ2A and AZ2B.

Statistic	AZ2A	AZ2B
Number of protein-coding genes	15,046	15,050
Total length of protein-coding gene (bp)	23,931,139	24,050,425
Average length of protein-coding gene (bp)	1,591	1,598
Total exon length (bp)	18,480,758	18,573,258
Number of exons	69,453	69,499
Average length of exon (bp)	266	267
Genes with one more exon	12,872	12,883

### Mitochondrial genome assembly

Mitogenome of AZ2 was also assembled as in a previous study^45^. A multifasta file of *Puccinia striiformis* mitogenomes containing PST-78^46^, Pst-DK0911^37^, Pst93-210^47^, Psh93TX-2^47^ and CY32^48^ acted as the starting reference genome, and the mitogenome of AZ2 was assembled with PacBio HiFi reads using Canu v2.2^49^. The assembled mitogenome was annotated with the GeSeq^50^ web browser (https://chlorobox.mpimp-golm.mpg.de/geseq.html) and the MITOS^51^ web server using genetic code 4 (http://mitos.bioinf.uni-leipzig.de/index.py). Next, the tRNA genes were then further evaluated using tRNAscan-SE v2.0.9^52^. A graphical map of the mitogenome was drawn using mtviz (http://pacosy.informatik.uni-leipzig.de/mtviz). A complete circular mitogenome of AZ2 was assembled with a total size of 101,852 bp and a guanine-cytosine (GC) content of 31.44% (Fig. S1). In total, 14 protein-coding genes (atp6, atp8, atp9, nad1, nad2, nad3, nad4, nad4L, nad5, nad6, cox1, cox2, cox3 and cob) and 24 tRNAs were detected in the AZ2 mitogenome located on the direct strand.

### Chromosomal synteny analysis

**Fig. 3:**
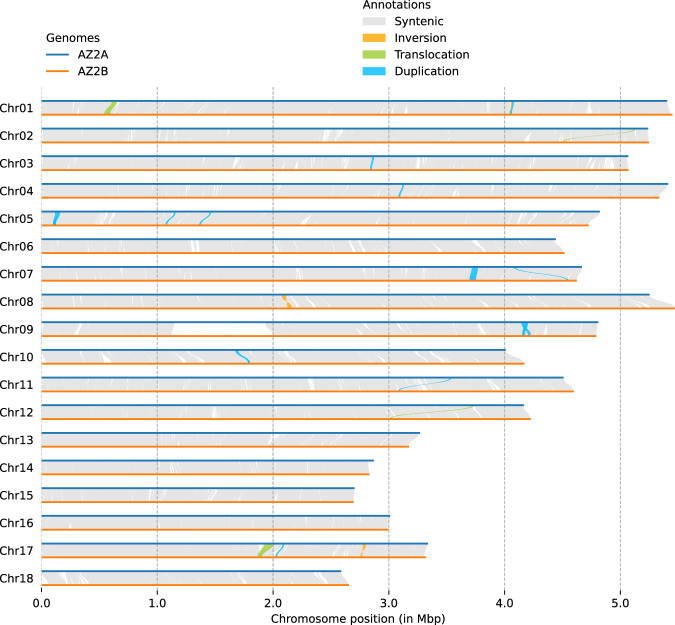
The sequence collinearity and structural variants between AZ2A and AZ2B. The haplotype AZ2A is used as the reference sequence and the haplotype AZ2B is the query. Collinear regions between the two haplotypes are shown by gray lines.

To investigate differences between the two haplotypes, the command nucmer in MUMmer v4.0^53^ with the parameters ‘–maxmatch -c 100 -b 500 -l 50’ was used for whole-genome alignments, and the alignment results were filtered using the command delta-filter with the parameters ‘-m -i 90 -l 100’. After format conversion with the command show-coords, SyRI v1.6.3^54^ using the default parameters detected the syntenic regions and structural variations. Plotsr v1.1.1^55^ was used to visualize the variations (Fig. 3). A total of 1128 syntenic regions with a cumulative size of 142.48 Mb (94.05%) were detected, indicating a high similarity between the two haplotypes. Furthermore, 227 translocations with a cumulative size of 1.70 Mb (~1.12%), 8 inversions with a cumulative size of 0.18 Mb (~0.12%), and 2778 duplications with a cumulative size of 5.02 Mb (~3.31%) were also detected.

## Data Records

All raw sequencing data and genome assembly of *Pst* isolate AZ2 have been deposited in the National Center for Biotechnology Information (NCBI) under BioProject ID PRJNA1025922 and PRJNA1026770. The PacBio HiFi, Hi-C, Illumina sequencing reads and RNA sequencing reads of AZ2 have been deposited in the NCBI Sequence Read Archive database with accession group numbers SRP465535^56^. All raw sequencing data of A153 and XZ-2 have been submitted to the NCBI Sequence Read Archive database (SRR26345460^57^ and SRR26345461^58^). Genome assembly is available from GenBank in the NCBI with accession number GCA_039519205.1^59^ and GCA_039519225.1^60^. The genome assembly and gene annotation results were also deposited in the figshare database^61^.

## Technical Validation

### Evaluation of the assembled genome

**Fig. 4:**
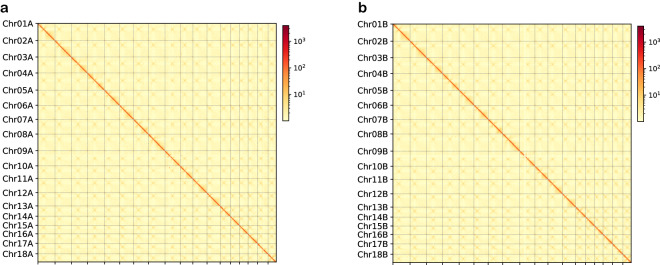
Heatmap of genomic interactions (with a resolution of 20 kb) of AZ2A (**a**) and AZ2B (**b**) chromosomes using Hi-C data. The strength of the interaction was represented by the color from yellow (low) to red (high).

The quality of genome assembly was evaluated using multiple methods. First, the accuracy of the Hi-C based chromosome construction was evaluated by chromatin contact matrix using HiC-Pro v3.0.0^62^, and contact maps were plotted with hicPlotMatrix of HiCExplorer v3.7.2^63^. The interactive Hi-C heatmap confirmed the good continuity of genome assembly (Fig. 4). Second, the BUSCO analysis using the basidiomycota odb9 database (genome mode) was performed to assess genome completeness using BUSCO v3.0.2b^64^ with *Ustilago maydis* as the reference species for Augustus gene prediction. The complete BUSCO scores (including single-copy and duplicated) of the two haplotypes accounted for 95.0% and 95.3%, respectively (Supplementary Table 3), suggesting good completeness of the genome assembly. Third, Illumina short reads and HiFi long reads from AZ2 were mapped to the assembly using BWA-MEM^65^ and minimap2 v2.24^66^, then QualiMap v2.2^67^ was used to evaluate the mapping quality. Mapping rates were > 96%, and sequencing coverage reached 99.99%, indicating good consistency between the diploid genome with Illumina and HiFi sequencing reads (Supplementary Table 4). Fourth, the consensus quality value (QV) and completeness of the genome were evaluated using Merqury v1.3^68^ with meryl v1.3 (under 19-mer) count. QVs for AZ2A and AZ2B, and shared AZ2A and AZ2B were 55.57, 59.02, and 56.96 (Genome accuracy > 99.999%), respectively (Table 4). The completeness scores for AZ2A and AZ2B were 92.15% and 92.23%, respectively. Finally, telomeres were annotated by searching for the CCCTAA or TTAGGG repeat sequences based on the method described previously^69^. In total, 34 of the 36 telomeres were detected on AZ2A, except for one telomere on chromosome 8 and one telomere on chromosome 16. Except for chromosome 9 on AZ2B containing one telomere, the other 17 chromosomes each contained telomere sequences at either end (Fig. 2, Supplementary Table 1). In general, this assembly can be described as a nearly telomere-to-telomere genome.

**Table 4:** Statistics of Merqury analysis for genome quality assessment.

Assembly	QV (quality value)	Error rate	Completeness (%)
AZ2A	55.57	2.77E-06	92.15
AZ2B	59.02	1.25E-06	92.23
both AZ2A and AZ2B	56.96	2.01E-06	97.52

### Evaluation of the gene annotation

The annotated and integrated proteins were also evaluated using BUSCO v3.0.2b^64^ with the basidiomycota odb9 database (protein mode). The complete BUSCO scores of the two haplotypes accounted for 97.7% and 97.9%, respectively, indicating high quality of the gene annotation (Table 5).

**Table 5:** Summary of BUSCO analysis of protein-coding genes in AZ2.

Statistic	AZ2A	AZ2B
Complete BUSCOs (%)	1304 (97.7%)	1307 (97.9%)
Complete and single-copy BUSCOs (%)	1245 (93.3%)	1250 (93.6%)
Complete and duplicated BUSCOs (%)	59 (4.4%)	57 (4.3%)
Fragmented BUSCOs (%)	20 (1.5%)	18 (1.3%)
Missing BUSCOs (%)	11 (0.8%)	10 (0.7%)
Total BUSCO groups searched	1335	

### Supplementary information

Supplementary information

## Data Availability

All sofware and pipelines used in this study were performed with the parameters described in the Methods section. If no detail parameters were mentioned for the sofware, default parameters were used as suggested by developer.
