# Supplementary material for: A fully haplotype-resolved and nearly gap-free genome assembly of wheat stripe rust fungus
Source: Sci Data. 2024 May 16;11:508. doi: 10.1038/s41597-024-03361-6 (PMC11099153; doi:10.1038/s41597-024-03361-6)
Supplement: Supplementary file 1 — Supplementary information [file 41597_2024_3361_MOESM1_ESM.doc]

**Supplementary information**

**
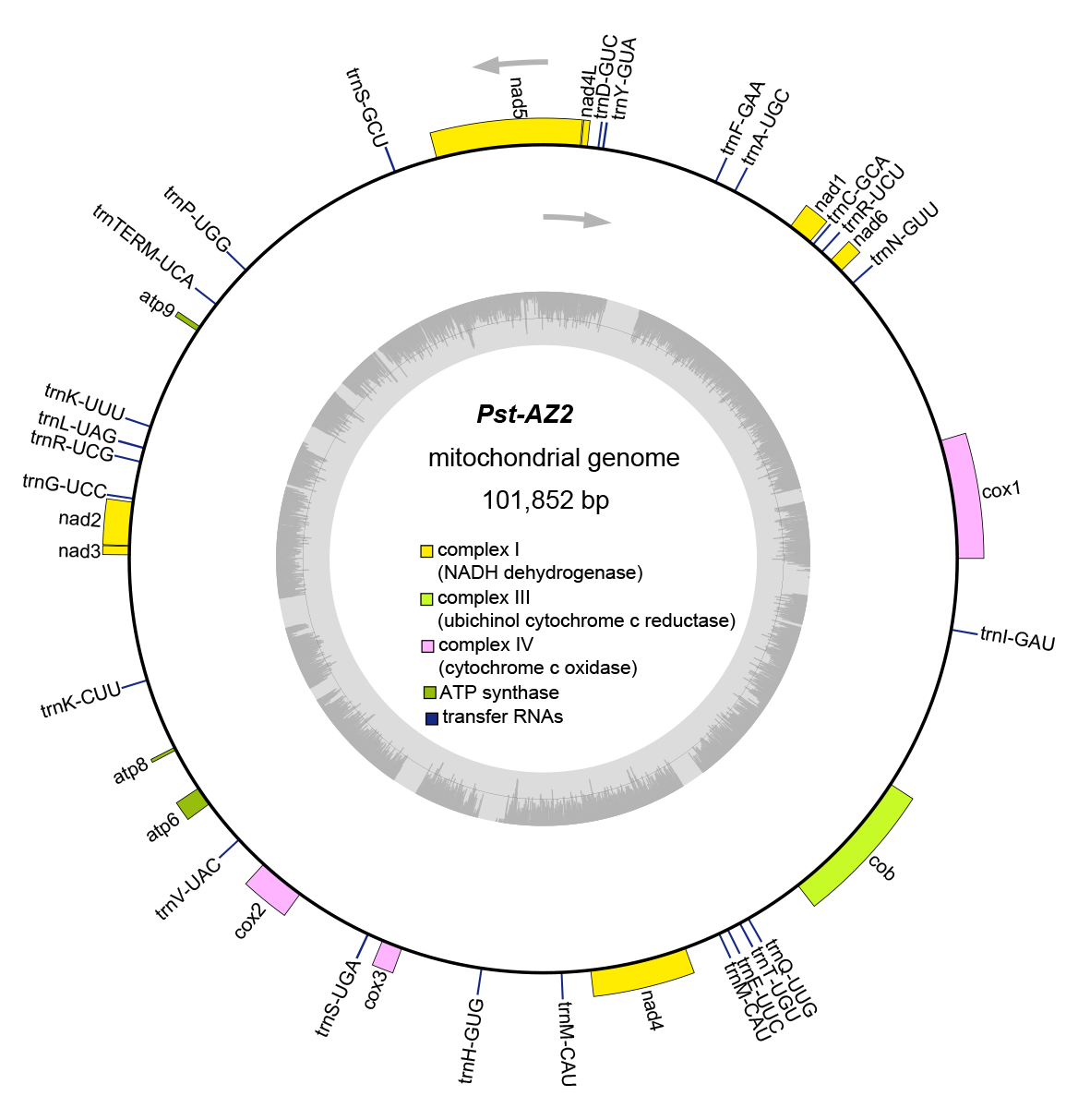
**

**Fig.S1.** The mitochondrial genome of *Puccinia striiformis* f. sp. *tritici* isolate AZ2.

**Supplementary Table 1. Statistics of chromosomal level assembly of *Puccinia striiformis* f. sp. *tritici* isolate AZ2.**

| Chr ID | Length (bp) | Contig number | Gap number | Gap length (bp) | Telomere number |
| --- | --- | --- | --- | --- | --- |
| Chr01A | 5,404,001 | 1 | 0 | 0 | 2 |
| Chr01B | 5,449,808 | 2 | 1 | 100 | 2 |
| Chr02A | 5,240,477 | 1 | 0 | 0 | 2 |
| Chr02B | 5,246,564 | 1 | 0 | 0 | 2 |
| Chr03A | 5,068,618 | 1 | 0 | 0 | 2 |
| Chr03B | 5,069,821 | 1 | 0 | 0 | 2 |
| Chr04A | 5,412,896 | 3 | 2 | 200 | 2 |
| Chr04B | 5,336,276 | 1 | 0 | 0 | 2 |
| Chr05A | 4,822,410 | 2 | 1 | 100 | 2 |
| Chr05B | 4,727,124 | 1 | 0 | 0 | 2 |
| Chr06A | 4,442,530 | 1 | 0 | 0 | 2 |
| Chr06B | 4,517,800 | 1 | 0 | 0 | 2 |
| Chr07A | 4,668,497 | 1 | 0 | 0 | 2 |
| Chr07B | 4,623,987 | 1 | 0 | 0 | 2 |
| Chr08A | 5,251,873 | 1 | 0 | 0 | 1 |
| Chr08B | 5,471,935 | 1 | 0 | 0 | 2 |
| Chr09A | 4,809,197 | 2 | 1 | 100 | 2 |
| Chr09B | 4,792,743 | 1 | 0 | 0 | 1 |
| Chr10A | 4,008,906 | 1 | 0 | 0 | 2 |
| Chr10B | 4,171,609 | 1 | 0 | 0 | 2 |
| Chr11A | 4,509,441 | 1 | 0 | 0 | 2 |
| Chr11B | 4,597,639 | 1 | 0 | 0 | 2 |
| Chr12A | 4,166,297 | 1 | 0 | 0 | 2 |
| Chr12B | 4,226,248 | 1 | 0 | 0 | 2 |
| Chr13A | 3,270,064 | 1 | 0 | 0 | 2 |
| Chr13B | 3,175,248 | 1 | 0 | 0 | 2 |
| Chr14A | 2,870,628 | 1 | 0 | 0 | 2 |
| Chr14B | 2,832,454 | 1 | 0 | 0 | 2 |
| Chr15A | 2,703,880 | 1 | 0 | 0 | 2 |
| Chr15B | 2,695,807 | 1 | 0 | 0 | 2 |
| Chr16A | 3,010,599 | 1 | 0 | 0 | 1 |
| Chr16B | 2,996,629 | 1 | 0 | 0 | 2 |
| Chr17A | 3,338,427 | 2 | 1 | 100 | 2 |
| Chr17B | 3,321,520 | 2 | 1 | 100 | 2 |
| Chr18A | 2,587,901 | 1 | 0 | 0 | 2 |
| Chr18B | 2,655,380 | 1 | 0 | 0 | 2 |
| Total | 151,495,234 | | | | |
| Mt | 101,852 | 1 | 0 | 0 | — |

**Supplementary Table 2.** Repeat annotations of AZ2 genome assembly.

|  | AZ2A | | | AZ2B | | |
| --- | --- | --- | --- | --- | --- | --- |
|  | Number | Length (bp) | Percent (%) | Number | Length (bp) | Percent (%) |
| DNA elements | 38,638 | 10,252,632 | 13.56 | 38,417 | 10,451,098 | 13.77 |
| Long interspersed nuclear elements (LINEs) | 563 | 203,111 | 0.27 | 711 | 247,603 | 0.33 |
| Long terminal repeats (LTRs) | 16,827 | 9,973,137 | 13.19 | 17,174 | 10,477,785 | 13.80 |
| Short interspersed nuclear elements (SINEs) | 46 | 20,200 | 0.03 | 114 | 10,342 | 0.01 |
| Unclassified | 30,843 | 7,913,674 | 10.47 | 25,626 | 7,445,085 | 9.81 |
| Satellite | 391 | 90,219 | 0.12 | 307 | 57,400 | 0.08 |
| Simple repeat | 422 | 100,980 | 0.13 | 864 | 373,953 | 0.49 |

**Supplementary Table 3.** Summary of BUSCO analysis of AZ2 genome assembly.

| Statistic | AZ2A | AZ2B |
| --- | --- | --- |
| Complete BUSCOs (%) | 1269 (95.0%) | 1272 (95.3%) |
| Complete and single-copy BUSCOs (%) | 1231 (92.2%) | 1233 (92.4%) |
| Complete and duplicated BUSCOs (%) | 38 (2.8%) | 39 (2.9%) |
| Fragmented BUSCOs (%) | 33 (2.5%) | 34 (2.5%) |
| Missing BUSCOs (%) | 33 (2.5%) | 29 (2.2%) |
| Total BUSCO groups searched | 1335 | |

**Supplementary Table 4.** Statistics of map rate and coverage of different types of sequencing reads.

|  | HiFi | Illumina |
| --- | --- | --- |
| Reads mapped (%) | 98.86 | 96.14 |
| Properly paired (%) | — | 95.90 |
| ≥1×(%) | 100 | 99.99 |
| ≥5×(%) | 99.97 | 99.92 |
| ≥10×(%) | 99.92 | 99.49 |
